# Assessment of the microvasculature in poppers maculopathy

**DOI:** 10.1007/s00417-021-05453-0

**Published:** 2021-11-20

**Authors:** T. Hamann, M. R. J. Wiest, M. Brinkmann, M. Toro, K. Fasler, J. Baur, K. B. Freund, Sandrine Zweifel

**Affiliations:** 1 Department of Ophthalmology, University Hospital Zurich, Frauenklinikstrasse 24, Zurich, 8091 Switzerland; 2 University of Zurich, Rämistrasse 71, Zurich, 8006 Switzerland; 3 Chair and Department of General and Pediatric Ophthalmology, Medical University of Lublin, Lublin, Poland; 4 Vitreous Retina Macula Consultants of New York, New York, NY USA; 5 Department of Ophthalmology New York University School of Medicine, New York, NY USA

**Keywords:** Poppers maculopathy, Multimodal imaging, OCT angiography, Drug toxicity, Retinal toxicity

## Abstract

**Purpose:**

To investigate a possible microvascular component of poppers maculopathy (PMP) using optical coherence tomography angiography (OCTA).

**Methods:**

Twelve patients suffering from poppers maculopathy were included. Health records, optical coherence tomography (OCT), and OCTA data was gathered and compared to a healthy control group (HC). PMP lesion type was determined by manifestation in OCT. OCTA-based evaluation of retinal vascular plexus and choriocapillaris (CC) was executed. Vessel density (VD) and vessel length density (VLD) in superficial and deep capillary plexus (SCP, DCP), as well as flow deficits (FD), within the foveal avascular zone (FAZ) in CC were assessed.

**Results:**

Median age of PMP patients was 40 (min 24; max 64) years, all male. Eleven patients presented with ellipsoid zone-type lesions; one patient showed a vitelliform-type lesion. No qualitative microvascular changes between PMP patients and HC were identified. Quantitative values for VD and VLD of SCP and DCP did not differ in between the two groups. The analysis of FDs in CC showed no deviation from PMP patients to HC.

**Conclusions:**

No vascular anomalies in qualitative and quantitative analysis in OCTA were detected in PMP patients. The constitution of the CC within FAZ of PMP patients does not differ from HC when assessed as FD.

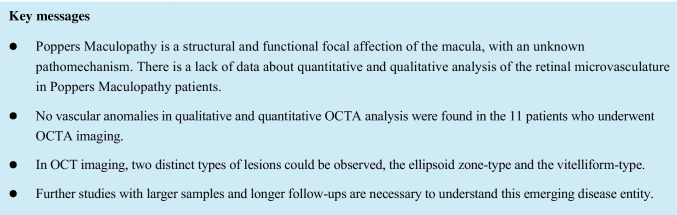

## Introduction

The term “poppers” describes drugs, which include volatile alkyl nitrites, which are consumed by inhalation of vapor. The effects include episodes of arousal, euphoria, and myorelaxation [1, 2]. Poppers maculopathy (PMP) refers to the functional affection of central vision and its structural manifestation as foveal alteration of the outer retinal architecture [2, 3]. Van Bol et al. [4] described three distinct phenotypes of PMP with various outcomes. The underlying pathogenesis of PMP is yet unknown. In the UK, 10% of the population had exposure to poppers, with higher levels of consumption in the “clubbing” collective and in gay men [1, 5, 6]. Within the population that used poppers in 2015 in the UK, 2.2% reported a drug-related affection of visual function, with an additional 10% who described a possible effect of poppers use on their eyesight [1].

Fluorescein angiography (FA) of eyes with poppers maculopathy typically shows either mild hyperfluorescence at the fovea or normal findings. No definitive signs of retinal vasculopathy have been identified with FA [2, 7–9]. Information about the microperfusion of the macula in PMP is scarce. One case report describing a 36-year-old patient with PMP did not show changes of flow signal in a qualitative assessment of optical coherence tomography angiography (OCTA), while another case report described an individual with choroidal flow deficits [10, 11]. Since the subfoveal cones affected in PMP lie in the foveal avascular zone (FAZ), the choriocapillaris (CC) constitutes their only blood supply. Conventional fluorescein angiography and indocyanine green angiography studies are limited to two-dimensional imaging and do not allow for selective analysis of specific retinal or choroidal layers such as the CC [12]. In order to overcome these limitations and to extend the understanding of PMP, qualitative and quantitative characteristics of macular, including CC microperfusion in a larger group of PMP patients using OCTA imaging, is executed in this study.

## Methods

This study was designed as a retrospective review of OCTA data acquired in patients with PMP. Twelve individuals with PMP were identified (Table 1), of whom 11 had gradable OCTA studies. The presented cases were assessed at the University Hospital of Zurich (USZ) and supplemented by an additional case from the Vitreous Retina Macular Consultants of New York. Institutional review board approval was obtained by the Western Institutional Review Board and Kantonale Ethikkommission Zurich (BASEC-nr 2019–0243). The study conformed to the tenets of the Declaration of Helsinki. All patients gave written informed consent to process and publish their data. Spectral-domain-OCT images were acquired on the Heidelberg Spectralis system (version 1.9.10.0) and assessed with Heidelberg software (Spectralis Viewing Module 6.0.9.0; Heidelberg Engineering). Phenotype of PMP lesion was determined according to Van Bol et al. [13]. Microperimetry was performed using the MAIA Microperimetry device (MAIA; CenterVue, Padova, Italy).

**Table 1:** Characteristics of poppers maculopathy (PMP) patients; extent of exposure to poppers and time from last exposure to examination are given as far as the information is available. Based on definition of PMP lesion phenotype by Van Bol et al. [4], 11 patients showed a disturbance of ellipsoid layer phenotype. In one patient, vitelliform lesion type was detected; in the majority of cases, visual acuity (VA) increased over the course of follow-up; one patient had a history of previous bilateral optic neuritis

ID	Age (a)/sex	OCTA	Duration/frequency of exposition upon first consultation	Time from first symptoms to examination	Time from exposure to examination	Phenotype of PMP	Follow-up duration	Termination of poppers use	Continuous EZ in follow-up	Initial VA OD; OS	Last VA OS; OS	Remarks
P01	39/m	Yes	Unknown/once per week	2 weeks	3 days	Disturbance of ellipsoid layer	1 months	Yes	Yes	20/20; 20/20	20/20; 20/20	Discontinuation of poppers use between follow-ups
P02	41/m	Yes	Four months/once or twice per week	2 days	3 days	Disturbance of ellipsoid layer	40 months	No, reduced to once per month	No	20/32; 20/32	20/32; 20/32	History of repeated affection of VA after poppers application
P03	42/m	No	15 years/2–3 × per month	7 days	8 days	Disturbance of ellipsoid layer	40 months	No	Yes	20/20; 20/32	20/20; 20/25	Known amblyopia of the left eye
P04	23/m	Yes	4 to 5 years/once per week	1 day	9 days	Disturbance of ellipsoid layer	3 months	Yes	Yes	20/25; 20/32	20/20; 20/20	None
P05	47/m	Yes	Unknown/unknown	6 years	2 months	Disturbance of ellipsoid layer	2 months	Yes	No	20/32; 20/32	20/32; 20/32	History of repeated affection of VA after poppers application
P06	64/m	Yes	40 years/once per week	No symptoms	One week	Vitelliform	None	No	n.a	20/20; 20/32	n.a.; n.a	Subjective affection of VA by sildenafil
P07	45/m	Yes	10 years/once peer week	No symptoms	One week	Disturbance of ellipsoid layer	None	No	n.a	20/20; 20/20	n.a.; n.a	None
P08	35/m	Yes	Unknown/unknown	Unknown	3–4 days	Disturbance of ellipsoid layer	6 months	Unknown	Yes	20/32; 20/25	20/25; 20/20	Status after bilateral optic neuritis*
P09	24/m	Yes	Unknown/unknown	5 days	6 days	Disturbance of ellipsoid layer	1 week	Unknown	No	20/32; 20/25	20/32; 20/25	None
P10	38/m	Yes	10 to 15 years/once per week	6 months	2.5 months	Disturbance of ellipsoid layer	6 months	Yes	Subtotal	20/30; 20/25	20/25; 20/20	None
P11	41/m	Yes	3 to 4 years/unknown	Unknown	Unknown	Disturbance of ellipsoid layer	6 weeks	Yes	No	20/32; 20/32	20/32; 20/32	None
P12	37/m	Yes	Unknown	2 months	Unknown	Disturbance of ellipsoid layer	None	Unknown	n.a	20/32 20/32	n.a. n.a	None

^*^VA 4 weeks before PMP RE = 20/40; LE 20/20, upon consultation for PMP no relative afferent pupillary defect, red desaturation or painful ocular motility were detected. In three patients, no information about continuity of ellipsoid zone (EZ) in follow-up was available (*n.a.* not available)

OCTA imaging was obtained in eleven patients with a swept-source-OCTA device (PlexElite 9000, Carl Zeiss Meditec AG, Munich, Germany). OCTA images were evaluated qualitatively and quantitatively and compared with age and sex matched healthy controls (*n* = 11). Following automatic projection artifact removal (PAR) (Carl Zeiss Meditec AG, Munich, Germany) *en face* projections of flow signal in the retinal superficial and deep capillary plexus (SCP, DCP) were created. Quality control of every image, including confirmation of accurate segmentation of retinal layers and an absence of projection and motion-artifects was performed by three independent investigators (SZ, MW, TH).

Quantitative analysis of vessel density (VD) and vessel length density (VLD) was performed for *en face* with signal strength values of ≥ 8. Subsequent analysis of VD and VLD was executed with ImageJ using a threshold algorithm for binarization as formerly reported [14, 15]. VD was measured as the proportion of the retinal sector occupied by vessels. VLD was assessed as the complete length of the skeletonized vessels using 1-pixel centerline extraction of the retinal vessels.

**Fig. 1:**
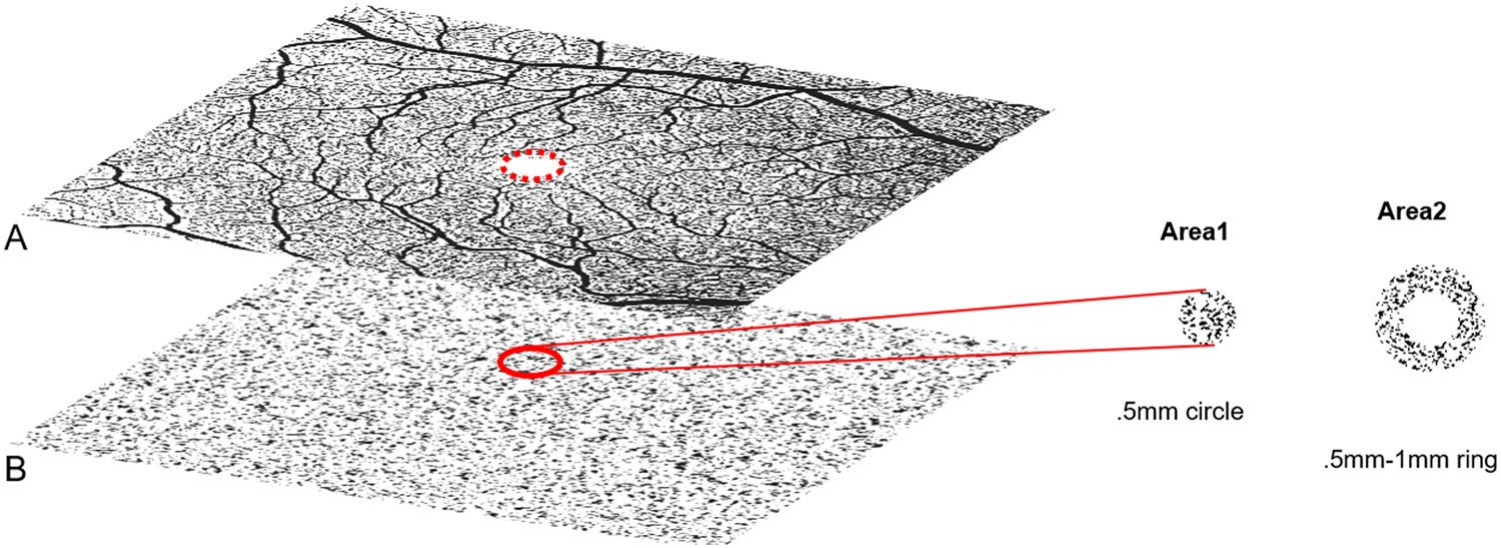
Quantification of flow deficits (FD) in the CC: **A** binarized 6 × 6 mm OCTA slab of the superficial capillary plexus with dotted red ring marking the FAZ; **B** binarized 6 × 6 OCTA slab of CC with a thickness of 20 μm and an offset of 29 μm below the retinal pigment epithelium (RPE). The red ring lines out the projected FAZ to the CC, which is measured as a .5 mm circle for quantification of FDs in CC = Area1, constituting an area of 0.196 mm2 as a standardized FAZ for all evaluated individuals. The remaining Area2 measure the FDs of perifoveolar ring

Choriocapillaris (CC) flow deficits (FD) were assessed as the fraction of the area without any flow signal in binarized choriocapillaris segmentation slabs (Fig. 1). This CC analysis was performed on using *en face* OCTA slab with a thickness of 20 µm and offsets 9 μm and 29 μm below the automated retinal pigment epithelium (RPE) segmentation. Binarization was performed with ImageJ (National Institutes of Health, Bethesda, MD, USA), using a threshold algorithm as previously described [14, 15]. The analyzed areas of interest were a circle with a diameter of 0.5 mm (area1), centered on the fovea and 1 concentric ring (area2) with an inner radius of 0.5 mm and an outer radius of 1 mm (Fig. 1). Area 1 comprised a scanned circle of CC slab, centered on the fovea, with a diameter of 0.5 mm and an area of 0.196 mm^2^, meant to analyze the CC FDs within a standardized FAZ. Average FAZ in men assessed in OCTA was described by Shiihara as an area of 0.274 ± 0.097 mm^2^ in healthy volunteers with an average age of 35.5 ± 9.6 years [16].

## Results

**Fig. 2:**
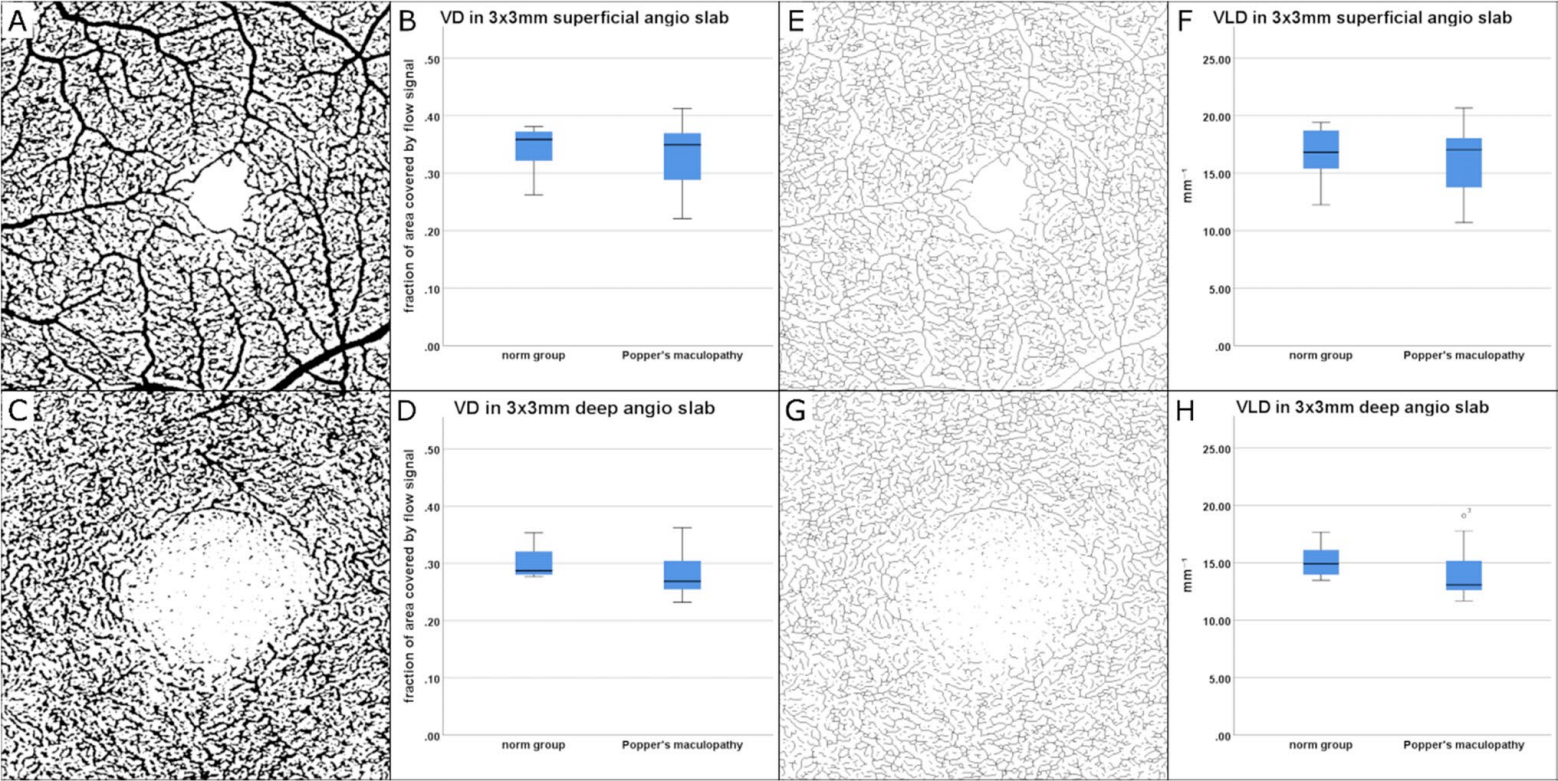
Quantitative findings of OCTA imaging in poppers maculopathy (PMP) patients (*n* = 11) compared with age and gender matched healthy controls (*n* = 11), (**A**–**H**). Vessel density (VD, as fraction of area covered by flow signal) of superficial and deep capillary plexus (SCP and DCP) was assessed in binarized OCTA images on the respective plexus (**A**, **C**) and visualized in box plots (**B**, **D**). No significant change was detected between the two groups. Vessel length density (VLD, mm^−1^) of SCP and DCP measured on skeletonized images (**E**, **G**) of the individual plexus did not show any significant difference between the PMP and the healthy group (**F**, **G**)

Twelve male patients with PMP and a median age of 40 years (range 24 to 64 years) were identified (Table 1). OCTA imaging was available in eleven of twelve PMP patients. Median follow-up, available in 9 patients, was 3 months (range 1 week to 40 months). Seven patients were HIV positive, under anti-retroviral treatment. Eleven patients demonstrated the subfoveal disturbance of ellipsoid zone-type lesions. One patient had a vitelliform-type PMP lesion. No macular-hole type PMP lesions were detected. The qualitative OCTA analysis of retinal flow signal in the SCP, DCP, and CC did not demonstrate any differences between PMP eyes and those of age and sex matched healthy individuals. Quantitative values for VD and VLD of SCP and DCP were well within range of age and sex matched healthy probands (Fig. 2). Similarly, quantitative analysis of CC FDs showed no significant differences between PMP patients and healthy controls. More specifically, measurements of area 1, which assessed the CC FD in a standardized FAZ (0.196 mm^2^) (Fig. 1) did not show qualitative or quantitative changes in PMP patients in comparison to controls.

The qualitative and quantitative analysis of the surrounding CC in form of areas 2 to 5 did not show any observable difference to the norm population either. Furthermore, fluorescein and indocyanine green angiography were performed in two PMP patients and did not demonstrate any abnormal vascular changes.

**Fig. 3:**
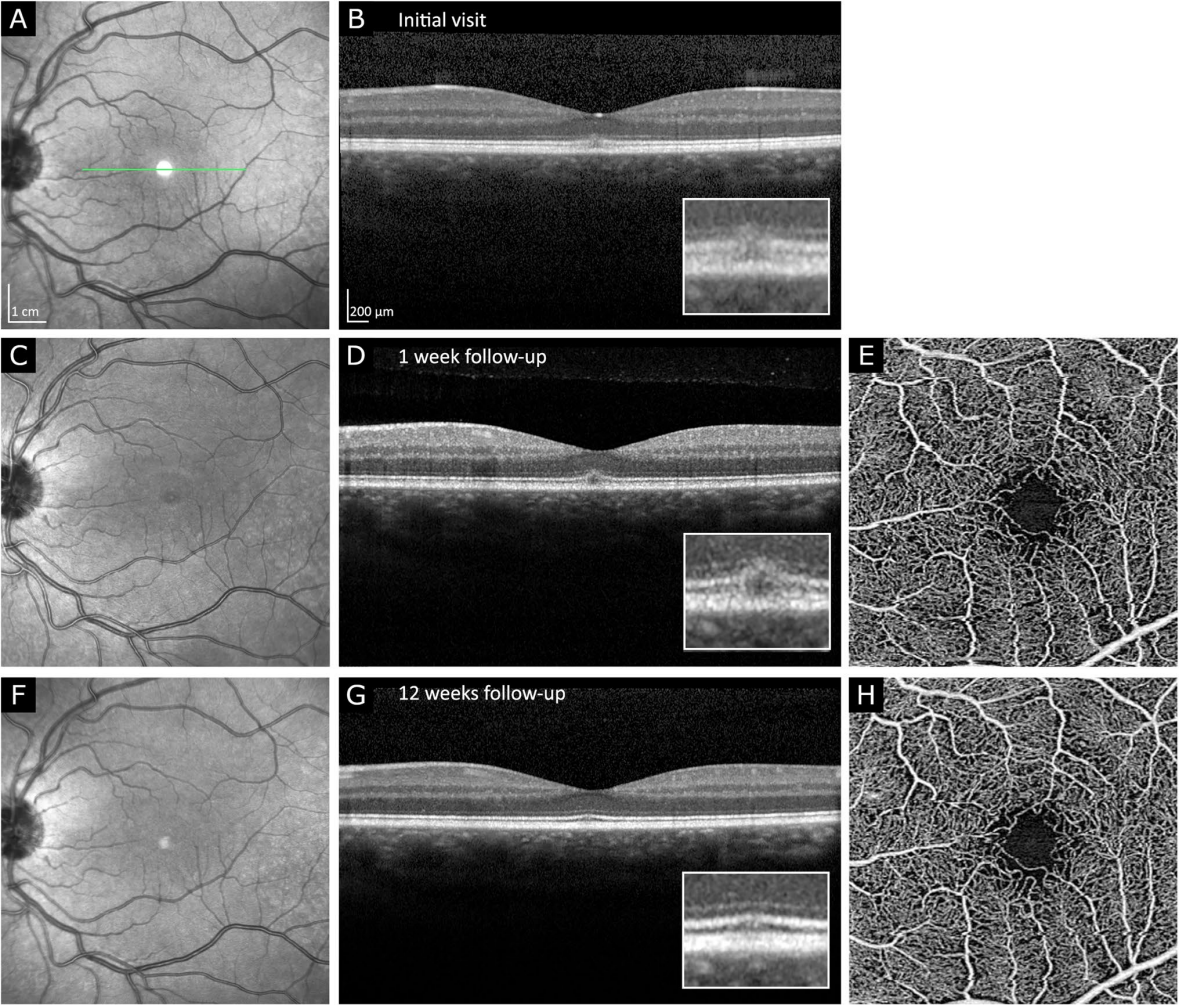
Resolution of ellipsoid zone-type PMP lesion over the course of 12 weeks in OCT imaging (**A**–**D**, **F**, **G**). Initial presentation (**A**, **B**): note the subfoveal disruption of ellipsoid zone (EZ) and affection of interdigitation zone (IZ). External limiting membrane (ELM) appears continuous but slightly elevated. Similar findings are depicted in 1-week follow-up (**C**, **D**). Near infrared en-face imaging (**C**) demonstrates foveolar hyporeflectivity. After 12 weeks (**F**, **G**) sub-foveolar EZ and IZ are assessable as continuous hyperreflective bands but slightly less reflective than the signal of the temporal, nasal continuations of the respective bands. OCTA of superficial capillary plexus of the fovea at weeks 1 and 12 did not show any pathologies (**E**, **H**)

Five patients discontinued the use of poppers between initial examination and follow-up visits. Within these five patients, four showed reestablished continuous ellipsoid zones upon follow-up (four of four with subfoveal disturbance of ellipsoid zone-type lesion) (Fig. 3). In summary, no microvascular changes, neither quantitatively nor qualitatively, was identified in PMP patients.

## Discussion

Poppers maculopathy patients do not show vascular anomalies in qualitative and quantitative analysis in OCTA when compared to healthy controls. The constitution of the CC within in the FAZ, assessed as CC FD within the FAZ of PMP patients, did not show values outside of the range of the findings of healthy controls.

While conventional fluorescein angiography and indocyanine green angiography are limited to two-dimensional analysis and do not allow for selective analysis of CC [12], OCTA allows for three-dimensional analysis of flow signal of the parafoveal vascular arcade in form of SCP, DCP, and assessment of CC within the FAZ. Thereby, in order to determine whether PMP shows foveal and parafoveal changes in the microvasculature in vivo, currently OCTA is the most adequate tool. Still, OCTA imaging has a limited diagnostic range concerning detection of excessively high and low flow signals [17]; as a consequence, potentially altered flow signals within these characteristics were not assessed in our study. While our results, including additional fluorescein and indocyanine green angiography in two PMP patients did not confirm any microvascular changes in patients with PMP compared to healthy controls, the cause of the PMP characteristic lesions remains unknown. As described by Bral et al., alkyl nitrates, which are a component of poppers, upon oxidation induce nitric oxygen (NO), which in turn causes an increase in cyclic guanosine monophosphate (cGMP) [18, 19]. cGMP is crucial in the photo-transduction pathway that enables Calcium^2+^ (Ca^2+^) and Sodium^2+^ to permeate cell membranes, which results in its depolarization [18, 19]. Nevertheless, continuous high levels of intracellular cGMP and Ca^2+^ cause oxidative stress, leading to photoreceptor cell death [19]. Strikingly, in PMP patients, the retina apart from the subfoveal region appears to be remarkably normal. While the retina features high metabolic activity, this is especially true for the fovea that exhibits the highest density of photoreceptors, which are in continuous focus of the incoming light [20, 21].

Thereby, high levels of oxidative stress accumulate on photoreceptor level, which necessitates elaborate anti-oxidative mechanisms to counter reactive oxygen species (ROS) pressure on the fovea [22]. One anatomic hallmark of these anti-oxidative mechanisms, the macula lutea derives its name from the highest density of macular pigment, more precisely mesa-zeaxanthin, lutein, and zeaxanthin found anywhere in the human body [23, 24]. In PMP the limits of homeostatic capacity of the foveal cone receptors appear to be breached, one potential pathomechanism being excessive oxidative stress, which manifests structurally as disturbance of subfoveal architecture and functionally as reduced VA and sensitivity of visual field. Consequently, the fovea can be described as poppers sensitive. Whether this is due to the one layered blood supply via the choriocapillaris, the rest of the retina constitutes a dual blood supply that is not assessable by our data. The potential role of ROS in PMP remains to be validated in future studies, which could shed light on the impact of antioxidants as a prophylactic supplement in poppers users or as a therapeutic agent in PMP patients.

In concordance with previous studies, five patients demonstrated reconstitution of EZ and resorption of hyperreflective intraretinal material upon follow-up [4, 25, 26]. Still, in two cases, despite structural improvement in OCT and normal OCTA findings, functional deficiencies persisted in form of reduced VA (P02 and P10, Table 1) which is in accordance with previously published case descriptions [27]. Additionally, in P10 persistent decreased sensitivity of the central visual field of 24 db of the right eye and 22db of the left eye were assessed in microperimetry. (We hypothesize that at least in these two patients, the recovery of PMP, even after discontinuation (P10) or drastic reduction (P02) of poppers application, is not a restitutio ad integrum but a process that can lead to alterations, which are not accessible in OCT imaging.) As Fajgenbaum [28] pointed out, PMP in OCT resembles a photopic retinal injury. In order to determine whether the proposed healing mechanism of photopic retinal injury, which is recruitment of surrounding photoreceptors, is applicable in PMP, future cellular and histological studies are warranted. No concordant statement on whether the suggested pathomechanism of photosensitization in PMP is plausible can be derived from our dataset.

One major limitation of this study is lack of exact dosage-effect analysis. Poppers is not a medical product; thereby, it is not produced with standardized levels of concentrations or application regime of the active agent. In order to offer some overview about the spectrum of the included PMP patients’ exposition to poppers and its relative timeline, also with regard to symptoms and last application in relation to examination, PMP patient histories were assessed accordingly as outlined in Table 1. Still, the follow-up duration of our patients ranges from none to 40 months; thereby, it is difficult to draw conclusions concerning the natural history of PMP. Prospective studies with larger patient numbers are required to mitigate this problem. Overall, visual impairment from PMP does not seem to be severe, although severe enough for patients to seek immediate medical assistance [1, 2, 4, 7–11, 18, 25–27]. A limitation of the definition of PMP phenotypes by Van Bol [4] is the possible manifestation of acquired vitelliform maculopathy that could structurally mimic the vitelliform-type PMP without being associated with visual symptoms.

Although structural improvement was observed in a significant number of our cases, functional impairment remained in two cases indicating persisting damage despite discontinuation of poppers use.

Taking into account the young median age of patients with PMP, awareness of possible long-term consequences of poppers use should be increased. Further studies are warranted to histologically confirm our results.
